# Influence of preheating on mechanical and surface properties of nanofilled resin composites

**DOI:** 10.4317/jced.56469

**Published:** 2020-05-01

**Authors:** Ali-Atef Elkaffass, Radwa-Ibrahim Eltoukhy, Salwa-Abd-Elraof Elnegoly, Salah-Hassab Mahmoud

**Affiliations:** 1 Assistant Lecturer, Operative Dentistry Dept, Faculty of Dentistry, Mansoura University, Egypt; 2 Clinical Assistant Professor, Operative Dentistry Dept, Faculty of Dentistry, Mansoura University, Egypt; 3 Professor of Dental Biomaterial, Faculty of Dentistry, Mansoura University, Egypt; 4 Clinical Professor and chairman of Operative Dentistry Dept, Faculty of Dentistry, Mansoura University, Egypt

## Abstract

**Background:**

Resin composite preheating is an innovative method that could be clinically beneficial by improving the handling properties, marginal adaptation, and surface properties of uncured nanofilled resin composite materials. There is conflict and unclear information regarding the effect of preheating on the microhardness, fracture toughness and surface roughness of nanofilled resin composites. Thus, it is important to assess whether dental clinicians can adopt preheating procedures without compromising composite mechanical strength. Objective: The purpose of this study was to evaluate the effect of preheating on microhardness, fracture toughness and surface roughness of nanofilled resin composite.

**Material and Methods:**

In this study, one commercial nanofilled resin composite Filtek Z350 XT was used. A total of 28 disc-shaped specimens were fabricated in a Teflon mold (10 mm diameter x 2 mm thick) for Vickers microhardness indentation test and surface roughness test. The samples were divided into two groups of 14 samples each, one group of samples was light-cured at room temperature (24ºC) without preheating (non-heated group), and the other group was light-cured after preheating (preheated group). Vickers hardness measurements of 14 specimens (n=7) either preheated or non-heated of the top and bottom surfaces was measured by means of microhardness tester by applying 100 g load for 10 s. Surface Roughness measurements (Ra) were obtained from 14 specimens (n=7) either preheated or non-heated with the atomic force microscope. Fourteen single-edge-notched-beam specimens were prepared for fracture toughness test (n=7) either preheated or non-heated with measurements (2.5 x 5 x 25 mm3) and a crack 2.12 mm in length. The specimens were tested via three-point bending mode, using a universal testing machine at crosshead speed of 1.0 mm/min until failure occurred.

**Results:**

Independent sample t- tests revealed no significant difference between non-heated and preheated groups for all tests (*p*>0.05). However, for Vickers hardness test, there were significant differences between top and bottom surfaces for non-heated and preheated groups (*p*<0.05). Moreover, surface roughness average Ra (nm) mean values of preheated group was higher than non-heated group but no significant difference between them was found (*p*>0.05).

**Conclusions:**

Preheating procedure did not negatively affect microhardness, fracture toughness and surface roughness of nanofilled resin composites so preheating is recommended for the other potential clinical advantages.

** Key words:**Preheating, nanofilled composites, microhardness, fracture toughness, surface roughness.

## Introduction

Preheating restorative resin composites has gained popularity among dental clinicians to ameliorate handling of composite material during placement and carving process (1). Preheating resin composites have a significant effect in the polymerization of multifunctional monomers which are the prime component of methacrylate-based dental restorative materials (2). Furthermore, free radicals and monomers mobility has been enhanced by increasing polymerization temperature and as a consequence a higher overall conversion occurs, which in turn results in improved mechanical, physical and surface properties of preheated composites, such as higher fracture toughness and enhanced surface hardness (3).

Composition and microstructure are accounTable for mechanical properties of resin composites (4). Adequate clinical performance together with enhanced mechanical properties of resin composites have made them more suiTable for posterior restorations (5). In spite of enhanced mechanical properties, mass fracture is considered one of two main concerns of composite restorations, the other being secondary decay (6). Hence, practitioners may consider preheating resin composite not only for increasing handling characteristics, but also with the expectation that mechanical properties will improve (7).

Therefore, mechanical properties of preheated resin composites should be evaluated to comprehend the effect of heat on the ability of resin composite material to resist fracture and wear as well against the forces of mastication. In previous studies (8,9), the outcomes associated with the investigation of this topic are unclear, and sometimes conflicting. Munoz *et al.* (8) mentioned that preheating resin composites may improve their hardness via greater monomer conversion. Conversely, Osternack *et al.* (9) suggested that preheating or precooling procedures has no effect on resin composite hardness.

Deb *et al.* (10) reported that preheating of the studied resin composites results in higher flexural strength. However, two more studies (11,12) show no difference in flexural strength between either preheated or non-heated composites. Aforementioned studies show that depending on composite type and different compositions of composites, preheating results in different effects on the mechanical properties of resin composites (13).

However, available data about the impact of composite preheating on microhardness, surface roughness and fracture toughness are scarce, and still inconclusive. Therefore, the purpose of the current study was to investigate the influence of composite preheating on three mechanical properties (microhardness, surface roughness and fracture toughness) of a conventional Nano-filled resin composite. Thus, the formulated null hypothesis was that mechanical properties would not show significant differences among preheating.

## Material and Methods

In this study, one commercial nanofilled resin composite Filtek Z350 XT (3M ESPE, St Paul, MN, USA) in the shade of A2 was used (Table 1). A total of 28 disc-shaped specimens were fabricated in a Teflon mold (10 mm diameter x 2 mm thick) for Vickers microhardness indentation test and surface roughness test. The samples were divided into two groups of 14 samples each, one group of samples was light-cured at room temperature (24ºC) without preheating (non-heated group), and the other group was light-cured after preheating (preheated group).

**Table 1:**
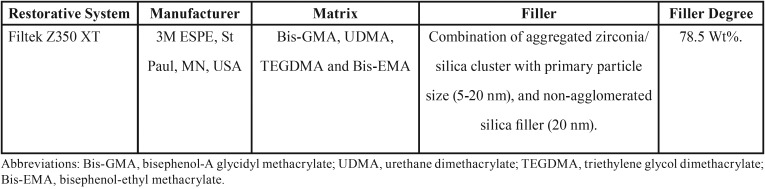
Materials used in the study.

For preheating resin composite prior to placement, a device called Therma-flo TM composite warming kit (Vista, Wisconsin, USA) was used according to manufacturer’s instructions. The warming device was operated for 30 min until it reached 68ºC and then the syringe tube is placed inside a heating chamber for 5 min to reach the temperature of the warming device. The syringe then removed from the device and then resin composite was applied immediately in one increment inside the mold.

For the preparation of specimens, the mold was placed on mylar strip on a glass slab and then was filled with resin composite and packed with gold-plated instrument under low light conditions. Subsequently, the resin composite was covered with another mylar strip and pressed with a glass slide to extrude excess material. The specimen was light-cured in close contact with its surface through the top mylar strip for 10 s. with a light emitting diode unit (Monitex BlueLEX™ GT-1200, New Taipei City, Taiwan). The wavelength of the unit measured between 450 and 500 nm. Light intensity was 1200 mW/cm2 in a normal mode, as measured by a radiometer (Optilux Radiometer Model 100, SDS Kerr, Danbury, CT, USA). The Teflon molds, glass slabs, clear mylar strips and gold-plated instruments were all warmed to 37°C before insertion of the resin. The specimens were polished with a sequence of 800, 1200 and 2000 grit silicon carbide paper under wet conditions and stored in distilled water in an incubator at 37˚C for 24 h prior to testing. Finishing and polishing was performed using Enhance and PoGo kits (Dentsply Caulk, Milford, DE, USA).

-Microhardness test

Vickers hardness measurements of 14 specimens (n=7) either preheated or non-heated of the top and bottom surfaces was measured by means of microhardness tester (Tukon 1102, Buehler, Uzwil, Switzerland) by applying 100 g load for 10 s. Three indentations with the random distance of 1 mm were taken for each surface and a mean value was calculated.

-Surface Roughness test

Surface Roughness measurements (Ra) were obtained from 14 specimens (n=7) either preheated or non-heated tested with the atomic force microscope (Autoprobe CP, Thermo-microscopes, Veeco Digital Instruments, Santa Barbra, Calif., USA). Imaging and scanning were performed in dry conditions and controlled temperature in the laboratory atmosphere. The basic operating principles have been reported elsewhere (14). The surface morphology of specimens was probed using sharp silicon nitride tips in ‘contact’ mode using cantilevers with a constant spring of about 7 to 10 N. The specimens were mounted with cyanoacrylate adhesive on a piezoceramic tube that provided three-dimensional movement of each sample with sub nanometer accuracy. As the specimen was scanned at constant force, the three-dimensional motion of the piezoceramic tube was recorded as an image and matched to the surface morphology. The 20-nm in-plane resolution of the AFM is dictated by the radius of curvature of the tip, while the vertical resolution is 0.1 nm. AFM images were collected at a very low scan rate of 1 Hz to obtain details of composite specimens and to avoid tip damaging. Five different areas were selected randomly with a scan area of 25×25 μm to obtain images with simultaneous deflection and height-mode images with a resolution of 512×512 pixels. Images were analyzed using dedicated software (Nanoscope v616r1, Veeco Metrology Group and WSxM 4.0 Develop 11.1, Nanotec Electronica, TreaCantas, Spain). The results of Ra are expressed as the means ± SD.

-Fracture toughness test 

Fourteen single-edge-notched-beam specimens were prepared for fracture toughness test (n=7) either preheated or non-heated with measurements (2.5 x 5 x 25 mm3) and a crack 2.12 mm in length according to adapted ISO 20795-2 standard method (ASTM 2005). A custom-made Teflon split mold was used designed with a slot placed centrally in the mold extending until it’s mid-height, which enabled central location of the notch. Paraffin was used as a neutral lubricating agent before placing the uncured resin composite in the mold to facilitate specimen removal after polymerization. Resin composite was packed into the mold supported with Mylar strip and glass slide. Polymerization was carried out for 40 s in five separated overlapping portions. The upper side of the mold was covered with Mylar strip and glass slide, before light polymerization. Light pressure was applied to expel excess material and trapped air. The mold is then removed, and specimens were polymerized from opposite side. After polymerization, specimens were polished using 800 grit silicon carbide abrasive papers. Specimens were stored in dry conditions at 37ºC for 24 h before testing. The specimens were tested via three-point bending mode, using a universal testing machine (Instron Model 4201, Canton, MA, USA) at crosshead speed of 1.0 mm/min until failure occurred. The following formulas were used to calculate the Mode I fracture toughness (KIC):

KIC = Mode I fracture toughness; PQ = fracture load; B = specimen thickness; W = specimen width

-Statistical analysis

The Shapiro-Wilk test at a=0.05 was applied to confirm the normal distribution of the results. Also, the modified Levene test was applied to confirm the validity of equal variance assumptions of mean values. The obtained data was analyzed using independent sample t-test at a=0.05 (IBM SPSS Statistics 21.0 software, IBM Chicago, IL, USA).

## Results

**Figure 1:**
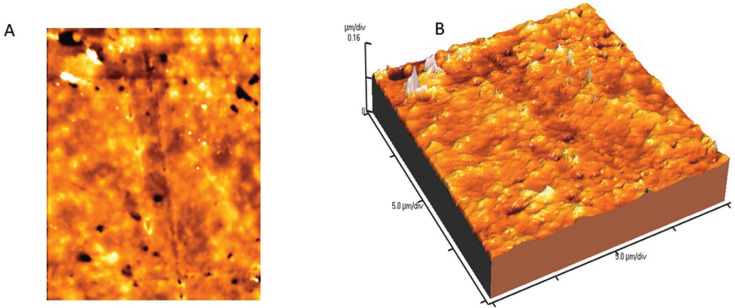
AFM image of non-heated Z350 XT resin composite (A) 2D image; (B) 3D image.

**Figure 2:**
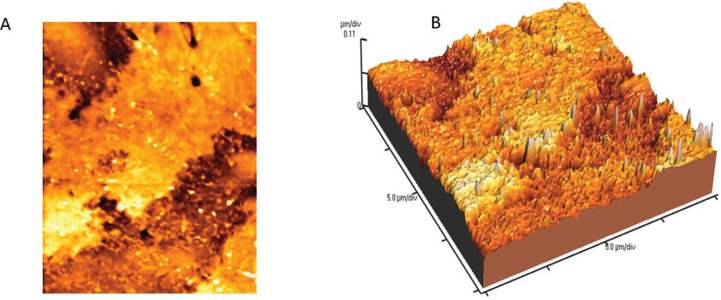
AFM image of preheated Z350 XT resin composite (A) 2D image; (B) 3D image.

Mean Vickers hardness (VHN), fracture toughness (MPa) and surface roughness average Ra (nm) mean values are presented in  Table 2- Table 4. Shapiro-Wilk test showed that the means of preheated and non-heated groups for all tests followed a normal distribution pattern (*p*>0.05). Also, the modified Levene test confirmed the validity of equal variance assumptions of mean values (*p*>0.05). Independent sample t- tests revealed no significant difference between non-heated and preheated groups for all tests (*p*>0.05). However, for Vickers hardness test, there were significant differences between top and bottom surfaces for non-heated and preheated groups (*p*<0.05). Moreover, surface roughness average Ra (nm) mean values of preheated Z350 XT resin composite (Fig. 1) was higher than non-heated Z350 XT resin composite (Fig. 2) but no significant difference between them was found (*p*>0.05).

**Table 2:**
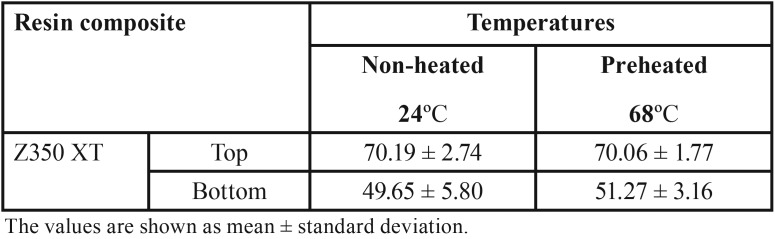
Vickers hardness (VHN) mean values (Standard deviations) achieved in non-preheated and preheated modes.

**Table 3:**
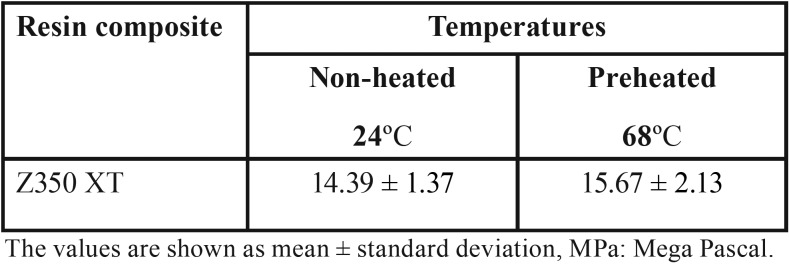
Fracture toughness (MPa) mean values (Standard deviations) achieved in non-preheated and preheated modes.

**Table 4:**
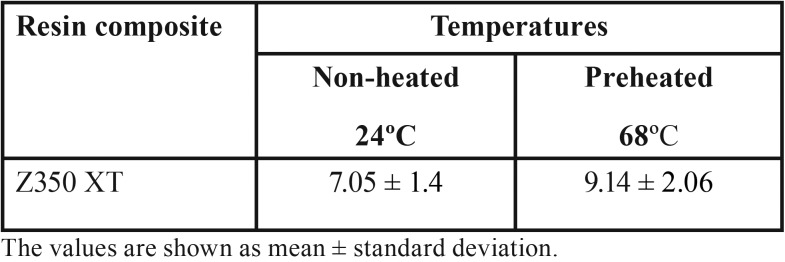
Surface roughness average Ra (nm) mean values (Standard deviations) achieved in non-preheated and preheated modes by AFM.

## Discussion

Studying preheating effect’s on mechanical properties provides valuable information to clinicians, to promote using packable resin composites in a flowable form. Thus, in a clinical situation, viscosity of packable resin composites is reduced upon preheating, offering a more flowable state which can be injected into cavity preparation rather than using conventional hand instruments for resin composites manipulation (10). Therefore, warm composite technique guarantee handling properties similar to flowable composite, gaining the advantages of outstanding mechanical, wear and surface properties correlated with the use of packable resin composites (15).

Former studies revealed that elevating resin composite temperature upon curing results in higher hardness and degree of conversion (16,17). This was attributed to decreased viscosity of the resin composite upon preheating, enhancing free radicals mobility and an accretion in the collision frequency for nonreactive groups (17). However, this study revealed that the hardness of nanofilled resin composites were not affected by composite preheating technique.

Thus, residual stresses generated during preheating process is an important factor to be considered. These stresses are a sort of energy concentrated within the material bulk without implementation of an external load (18). It was already found that these residual stresses are elevated upon rising temperature of resin composites. However, it was expected that these stresses were probably released 48 hours after photo-polymerization (18).

Preheating temperature and the time between composite dispensing and light initiation should be considered. Therefore, stabilizing temperature until light-curing process is ultimate. Therefore, composite temperature was strictly standardized in the present study, as the insertion time to the mold was 40 secs and the curing time was 10 secs. However, these 50 secs may have reduced the resin composite temperature. Daronch *et al.* (19) reported 50 % drop in temperature within 2 min in the composite samples upon removal from the heating device. Therefore, those authors suggested that clinicians must work very quickly to ensure the least temperature drop possible when using a heating device for the best clinical performance (19).

Moreover, hardness measurement is an indirect method to evaluate the conversion of carbon double bonds in a resin composite. It has been shown that a bottom to top vicker’s hardness number of 80% is related to a bottom to top conversion of 90%. However, bouschlicher *et al.* (20) refused an accurate correlation between these two parameters. They also stated that the ratio of bottom to top degree of conversion is independent of resin composite formulation (20,21). Top surfaces of resin composite samples showed greater microhardness compared to bottom surfaces which can be explained by the fact that light is attenuated as it travels through composite upon light curing process. In the present study, specimens thickness’s was 2 mm which is the same thickness of composite placed in clinical situation using incremental placement technique (2,22). At a depth of 2 mm, the attenuation of light may reduce irradiance to approximately 75% of that reaching the top surface (22,23).

Fracture toughness is meaningful mechanical property for brittle materials, although the results cannot be extrapolated to the clinical behavior without considering some aspects, namely flaw distribution (24) and structural reliability of the material (25). Nonetheless, the *in vitro* fracture toughness test is recommended by the ISO 4049/2000 specification for polymer-based materials and is widely used for comparative purpose (26,27). The findings of the study showed that preheating nanofilled resin composites did not alter the fracture toughness.

This was in agreement with Froes-Salgado *et al.* (12) whom conducted a study that evaluated the effect of composite preheating on flexural strength of a nanofilled composite and found that preheating resin composite prior to light curing did not alter the flexural strength. Since preheating did not affect fracture toughness, we believe that the only factor that can affect the flexural strength of resin composites is the filler load. Uctasli *et al.* (11) suggested that the filler content of resin composites affect their mechanical properties, as resin composites with the higher filler content showed higher flexural strength and flexural modulus.

Therefore, a great variation was found in viscosity of different resin composite materials upon preheating, which was attributed to the wide variety in composition, chemistry and filler content of the currently used resin composites. Upon increasing molecular weight in addition to the high capability for hydrogen bonding, viscosity of resin composite will increase (28). Also, polymer chains turn into more entangled structure upon increasing filler content due to increased chain length and forming more side chain branches, resulting in higher viscosity resin composites (28). Likewise, these obstacles (chain entanglement and hydrogen bonding) can be overwhelmed by preheating procedure by giving sufficient energy to allow molecules freedom to move in a less hindered sheering pattern with respect to one another (28). In general, the filler surface contour, the filler loading level, and the filler size distribution influence the capability of particles to slide past one another easily (29).

The results of the current study showed no significant differences in surface roughness between non-heated and preheated nanofilled resin composite. However, preheated group showed slightly higher values. The nanofilled Filtek Z350 resin composite consists of both nanoparticles and nanocluster fillers 82% by wt. Nanoparticles are discrete non-agglomerated and non-aggregated silica and zirconia fillers of 20 nm and 4-11 nm in size (30). The nanocluster particles increase physical properties, filler loading and polish retention of the nanofilled composite. Thus, the uniform distribution of precured silica particles in the organic matrix is the reason of not being affected by the preheating procedure (30).

According to the limitations of this study, the null hypotheses stating that there is no significance difference in microhardness, fracture toughness and surface roughness between non-heated and preheated nanofilled resin composite, was totally accepted.

## Conclusions

Preheating procedure did not negatively affect microhardness, fracture toughness and surface roughness of nanofilled resin composites so preheating is recommended for the other potential clinical advantages.
